# Comparative analysis of the fecal microbiota of healthy and injured common kestrel (*Falco tinnunculus*) from the Beijing Raptor Rescue Center

**DOI:** 10.7717/peerj.15789

**Published:** 2023-08-22

**Authors:** Yu Guan, Lei Bao, Lei Zhou, Chang Dai, Zhisai Li, Shuai Zhang, Yugang Shang, Wenhui Niu, Yizhuo Zhang, Hongfang Wang

**Affiliations:** 1 Beijing Normal University, Beijing, China; 2 International Fund for Animal Welfare, Beijing Raptor Rescuer Center, Beijing, China; 3 Beijing Zoo, Beijing, China

**Keywords:** Common kestrel, Fecal microbiota, 16S rRNA gene, Wild raptor rescue

## Abstract

The gut microbiota is a complex ecosystem that interacts with many other factors to affect the health and disease states of the host. The common kestrel (*Falco tinnunculus*) is protected at the national level in China. However, the available sequencing data of the gut microbiota from the feces of wild common kestrels, especially for being rescued individuals by professional organization, remains limited. In the present study, we characterized the fecal bacterial communities of healthy and injured common kestrels, and compared the structure of their fecal microbiota by analyzing the V3–V4 region of the 16S rRNA gene using high-throughput sequencing technology with the Illumina MiSeq platform. We found that Firmicutes, Proteobacteria and Actinobacteria were the most predominant phyla in common kestrels. Further, the beta diversity analysis showed that changes in gut microbes were associated with injuries to the common kestrel. The Bacteroides/Firmicutes ratio was significantly lower in the injured group. At the genus level, *Glutamicibacter* showed significant difference in the two groups. The aim of our current study was to characterize the basic bacterial composition and community structure in the feces of healthy common kestrels, and then compare the differences in the fecal microbiota between healthy and injured individuals. Patescibacteria, Spirochaetes, and *Glutamicibacter* may be studied as potential biomarkers for certain diseases in raptors. The results could provide the basic data for additional research on the fecal microbiota of common kestrels and contribute to the rescue of wild raptors in the future.

## Introduction

Gut microbiota, as the complex microbial community, plays crucial roles in lots aspects of animal host, such as development, immunology and physiological functions (Malmuthuge, Griebel & Guan, 2015; Nicholson, Holmes & Wilson, 2005) and is also closely related to the diet, health and age of host (Barelli et al., 2015; van Dongen et al., 2013). With the rapid development and the lower cost of advanced sequencing technology, the data of gut microbiota from the avian has markedly increased in recent years (Waite & Taylor, 2015, 2014), but the amount of data still lags behind the other species, including mammals and insects (Ge et al., 2021; Hildebrand et al., 2013; Nicholson, Holmes & Wilson, 2005).

The relationship between gut microbiota and host health have been studied in many species, such as *Cuon alpinus*, *Lasiopodomys brandtii* (Bo et al., 2022; Sekirov et al., 2010; Wu et al., 2022, 2016; Xu et al., 2023). The gut microbiota is considered to be an important factor affecting the immune system and brain activity of host, and the imbalance of gut microbiota may lead to obesity and cardiovascular disease (Lee & Hase, 2014). Furthermore, under the principle of non-invasive sampling, researches into the exact health of animals are relatively inadequate, especially for the wild animals. In recent years, advanced sequencing technology is applied to characterize and evaluated the composition and structure of gut microbiota for wild animals in China, which mainly focused on the various mammals, such as giant panda (*Ailuropoda melanoleuca*) (Li et al., 2015), bharals (*Pseudois nayaur*) (Chi et al., 2019), rhesus macaques (*Macaca mulatta*) (Chen et al., 2020) and Asian black bears (*Ursus thibetanus*) (Su et al., 2020). Due to the difficulties of sampling, the available data of the gut microbiota of the wild raptors are still limited now. In our present study, the Beijing Raptor Rescue Center (BRRC) possesses complete legal qualifications to rescue and cure the wild raptors, which provides enough opportunities to study their gut microbiota, as well as the monitoring of health conditions.

Meanwhile, many researches have revealed that the composition and structure of gut microbiota of the avian may be influenced by population diversity and unique characteristics, such as the physiological attributes of fly ability and migratory behavior of birds (Colston & Jackson, 2016; Guan et al., 2020; Stanley et al., 2015; Winter, Johnson & Shaffer, 2006). However, most of the previous studies for the gut microbiota of the avian focused on the common species, including domestic poultry and parts of wild birds, such as chicken (Ding et al., 2017; Rychlik, 2020; Wang et al., 2017), hoatzin (*Opisthocomus hoazin*) (Godoy-Vitorino et al., 2012), kakapo (*Strigops habroptilus*) (Waite, Deines & Taylor, 2012), black-legged kittiwakes (*Rissa tridactyla*) (van Dongen et al., 2013), capercaillie (*Tetrao urogallus*) (Wienemann et al., 2011) and parrot (Xenoulis et al., 2010). Although raptors are the crucial parts of the ecosystem and food chain, the specific mechanism and function of the gut microbiota of raptors are still unclear. Compared with captive and breeding species, data on the basic composition and structure of the gut microbiota of raptors have so far been very limited.

The common kestrel (*Falco tinnunculus*) is a widely distributed medium-size raptor that belonging to the family *Falconidae*. It is reported that due to the special diet and life style, which composed mainly by small mammals and reptiles, such as rodents, the common kestrel is considered as a typical potential pathogen carrier (Aparicio, 2000; Village, 2010). Although previous researches on common kestrels are comparatively comprehensive, including macroecology (Aschwanden, Birrer & Jenni, 2005; Geng et al., 2009; Lihu et al., 2007) and molecular ecology (Padilla et al., 2009; Riegert, Fainová & Bystřická, 2010), there are few studies of the gut microbiota on common kestrel. For example, we first reported the bacterial composition and structure of the gut microbiota of a wounded common kestrel rescued by BRRC (Guan et al., 2020). Then, Zhou et al. (2020) have analyzed the gut microbial communities of the Eurasian kestrel at different developmental stages (Zhou et al., 2020). With the advantages of BRRC, we could combine the results of gut microbiota with the actual observation and measurements to provide the advanced methods for raptors monitoring.

The aim of our present study was to characterize the basic bacterial composition and community structure in the feces of healthy common kestrels, and then compare the differences between healthy and injured common kestrels in their intestinal bacterial communities. The results could provide the basic data for additional research on the fecal microbiota of common kestrels and contribute to the rescue of raptors in the future.

## Materials and Methods

### Sample collection

During June and August of 2019, we collected ten fresh fecal samples of common kestrels at the Beijing Raptor Rescue Center (BRRC). BRRC possesses complete legal qualifications to rescue and cure the wild raptors. All rescued raptors of prey live in special living areas that mimic the temperature and humidity of the wild. Fecal samples were collected from different healthy (*n* = 6; H1–H6) and injured (*n* = 4; D1–D4) common kestrels, which had been reared for less than 6 months (Table S1). All injured kestrels had traumatic injured, such as fractures, and were treated with multiple surgeries and drug therapies. The feces samples of common kestrel were all collected immediately after defecation using sterile containers and then stored at −80 °C in the laboratory for subsequent bacterial analysis of uncultured bacteria. This study was performed in accordance with the recommendations of the Animal Ethics Review Committee of Beijing Normal University (approval reference number: CLS-EAW-2019-027).

### DNA extraction and sequencing of 16S rRNA gene amplicons

DNA was extracted from fecal samples of common kestrels using an E.Z.N.A.® Stool DNA kit (Omega Bio-tek, Norcross, GA, USA) following the instructions. The quality of the extracted DNA was assessed using a Nanodrop 2000. The hypervariable V3 and V4 regions of the bacterial 16S rRNA gene were amplified using the primer 338F (5′-barcode-ACTCCTACGGGAGGCAGCAG-3′) and 806R (5′-GGACTACHVGGGTWTCTAAT-3′) (Wu et al., 2016). PCR was performed as follows: denaturation at 95 °C for 3 min, followed by 25 cycles at 95 °C for 30 s, 55 °C for 30 s, and 72 °C for 30 s, with a final extension at 72 °C for 5 min. PCR amplification was performed in triplicate, with each 20 μL reaction containing 4 μL of 5 × FastPfu Buffer, 2 μL of 2.5 mM dNTPs, 0.8 μL of each primer (5 μM), 0.4 μL of FastPfu Polymerase, and 10 ng of template DNA.

### Illumina MiSeq sequencing

The amplified PCR products were extracted from 2% agarose gels and purified using an AxyPrep DNA Gel Extraction kit (Axygen Biosciences, Union City, CA, USA) following the manufacturer’s protocols and then quantified using QuantiFluor™-ST system (Promega, Madison, WI, USA). Purified PCR products were pooled in equimolar amounts, and high-throughput sequencing was performed on an Illumina MiSeq PE300 platform following the standard protocols (Shanghai Majorbio Bio-pharm Technology Co., Ltd, Shanghai, China).

The raw data were submitted to the NCBI Sequence Read Archive (SRA) database under the accession number SRP293621.

### Processing of sequencing data

Raw paired-end reads were demultiplexed and quality-filtered using QIIME (Version 1.17) (Caporaso et al., 2010), after which the reads were truncated at any site with an average quality score <20 by a 50 bp sliding window. Reads that were shorter than 50 bp or contained ambiguous nucleotides were eliminated. No mismatches were allowed for exact barcode matching and two nucleotide mismatching was allowed in primer matching. Reads were merged based on their overlap, which was no shorter than 10 bp. Unassembled reads were discarded.

The sequences were clustered into operational taxonomic units (OTUs) at a 97% similarity level using UPARSE (version 7.1; http://drive5.com/uparse/) (Edgar, 2013). Chimeric sequences were removed using UCHIME. The 16S rRNA gene sequences were taxonomically analyzed using RDP Classifier (https://github.com/rdpstaff/classifier) against the SILVA (v 132) 16S rRNA database with a confidence threshold of 70% (Amato et al., 2013).

The data were analyzed using the free online platform of Majorbio Cloud Platform (www.majorbio.com).

### Data analysis

All alpha diversity indices, including Sobs, Chao1, ACE, Shannon, Simpson, and Good’s coverage, as well as the beta diversity were calculated and generated using QIIME (Version 1.9.1). To standardize everything, we randomly select the size of the smallest library sequences from each sample 1,000 times and calculate the average. The rarefaction curves, rank abundance curves, and stacked histogram of relative abundance were generated and displayed using R (Version 3.3.1; R Core Team, 2016).

The hierarchical clustering trees were built using unweighted pair-group method with arithmetic mean (UPGMA) based on weighted UniFrac distance matrices at phylum level. Principal coordinate analysis (PCoA) and nonmetric multidimensional scaling (NMDS) based on unweighted UniFrac distance matrices at OTU level were performed and displayed using QIIME (Version 1.9.1) and R (Version 4.1.3; R Core Team, 2022), as were hierarchical clustering trees and the heatmap of clustering for genera abundance. Permutational Analysis Of Variance (PERMANOVA) based on Bray-Curtis showed the significant differences between the groups. The Wilcoxon rank-sum test of alpha diversity and significance were applied and then displayed by R (Version 3.3.1).

## Results

### Overview of the sequencing data

A total of 542,310 high-quality reads were obtained from the raw sequencing data after quality control and optimization. These reads were then classified into 1,989 unique OTUs at a 0.97 sequence identity cutoff for 10 fecal samples from different common kestrels.

Alpha-diversity indices (Sobs, Shannon, Simpson, Chao1, ACE and Good’s coverage) are shown in Table 1. Rarefaction curves (Fig. S1) reached a relatively flat level, indicating that the amount of sequencing data was sufficient for further analysis.

**Table 1:** Alpha diversity of gut microbiota from healthy and injured common kestrels.

Sample	Sobs	Shannon	Simpson	Ace	Chao	Good’s coverage
H1	153.000	1.930	0.213	361.726	271.333	0.998
H2	118.000	0.505	0.822	251.473	185.105	0.999
H3	40.000	1.553	0.310	51.046	52.000	1.000
H4	85.000	1.383	0.382	149.908	112.067	0.999
H5	74.000	0.629	0.796	82.667	80.600	1.000
H6	99.000	0.329	0.902	134.658	124.500	0.999
Mean	94.833	1.055	0.571	171.913	137.601	0.999
D1	509.000	3.655	0.056	555.925	548.115	0.998
D2	276.000	1.291	0.589	354.393	334.672	0.998
D3	198.000	2.080	0.192	278.616	263.029	0.998
D4	242.000	3.226	0.065	442.741	374.000	0.998
Mean	306.250	2.563	0.226	407.919	379.954	0.998

### Bacterial composition and relative abundance

We detected 32 phyla, 78 classes, 204 orders, 362 families and 782 genera in the fecal microbiota of the 10 common kestrel.

For the healthy common kestrels, *Firmicutes* (50.21%) was the most predominant phylum, followed by *Proteobacteria* (47.99%), *Actinobacteria* (1.68%), *Cyanobacteria* (0.05%) and *Bacteroidetes* (0.04%). The Bacteroides/Firmicutes ratio of healthy common kestrels was 6.97 × 10^−4^. At the genus level, *Escherichia-Shigella* (36.38%), *Paeniclostridium* (21.92%), *Raoultella* (9.60%), *Clostridium_sensu_stricto_1* (8.74%) and *Enterococcus* (6.63%) were the top five genera in the healthy common kestrel group.

**Figure 1:**
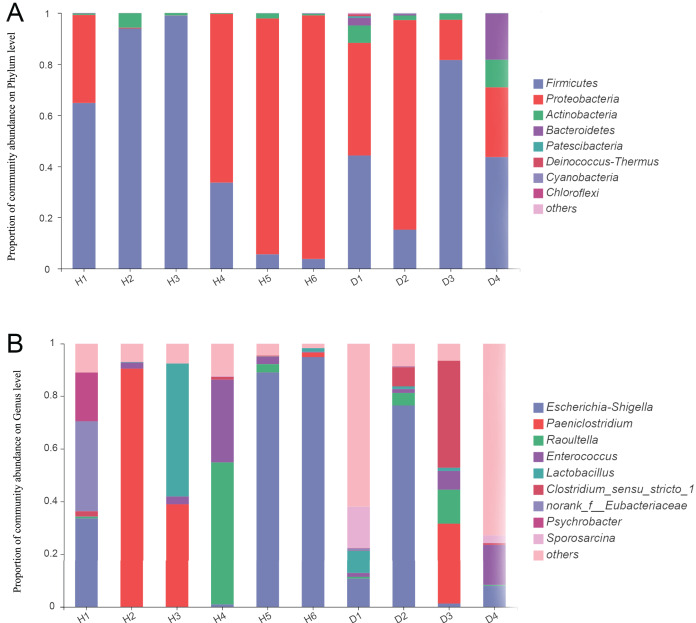
The bar plot of relative abundance in the gut microbiota of common kestrels at phylum (A) and genus (B) level. The horizontal axis represents the sample names, while the vertical axis represents the proportion of species in each sample. Different colored bars represent different species, and the length of the bars represents the relative proportion of each species. H means healthy group while D means disease group.

At the phylum level, *Firmicutes* (46.12%), *Proteobacteria* (42.33%) and *Actinobacteria* (5.46%) were also the most dominant phyla for injured common kestrels, the same as the healthy group, whereas *Bacteroidetes* (5.45%) and *Patescibacteria* (0.23%) were the fourth and fifth most common phyla. The Bacteroides/Firmicutes ratio of injured common kestrels was 0.12. *Escherichia-Shigella* (24.10%) was the most abundant genus in the injured group, followed by *Lactobacillus* (12.24%), *Paeniclostridium* (7.57%), *Enterococcus* (6.30%) and *Raoultella* (4.65%). To further assess the primary bacterial composition and the relative abundances of species, the proportion stacked bar plot at both the phylum (A) and genus (B) levels were generated and are shown in Fig. 1.

### Composition differences in fecal microbiota between healthy and injured common kestrels

**Figure 2:**
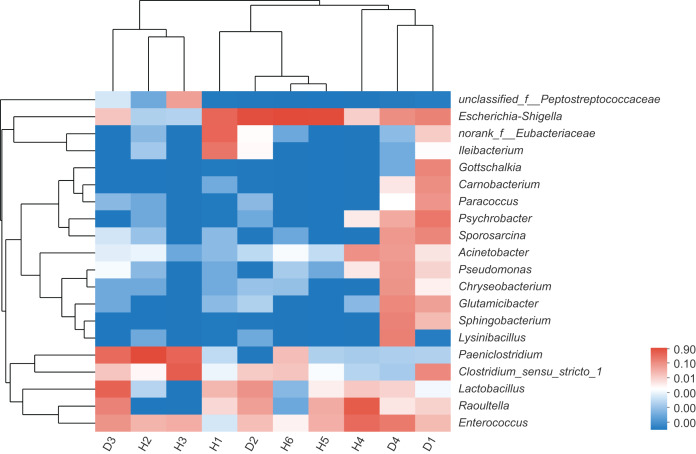
The heatmap of clustering for species abundance. The horizontal axis represents the sample names, while the vertical axis represents the species names. The abundance variation of different species in each sample is displayed using color gradients. The numerical values corresponding to the color gradient are shown on the right side of the graph. H means healthy group while D means disease group.

To assess the differences in the bacterial community structures between samples, hierarchical clustering trees were generated using UPGMA with weighted UniFrac distance matrices at phylum level. However, no significant grouping or aggregation between the healthy and the injured samples was observed. Subsequently, community heatmap analysis at genus level was conducted, and the results are shown in Fig. 2, which showed that the healthy and the injured samples were grouped together separately.

**Figure 3:**
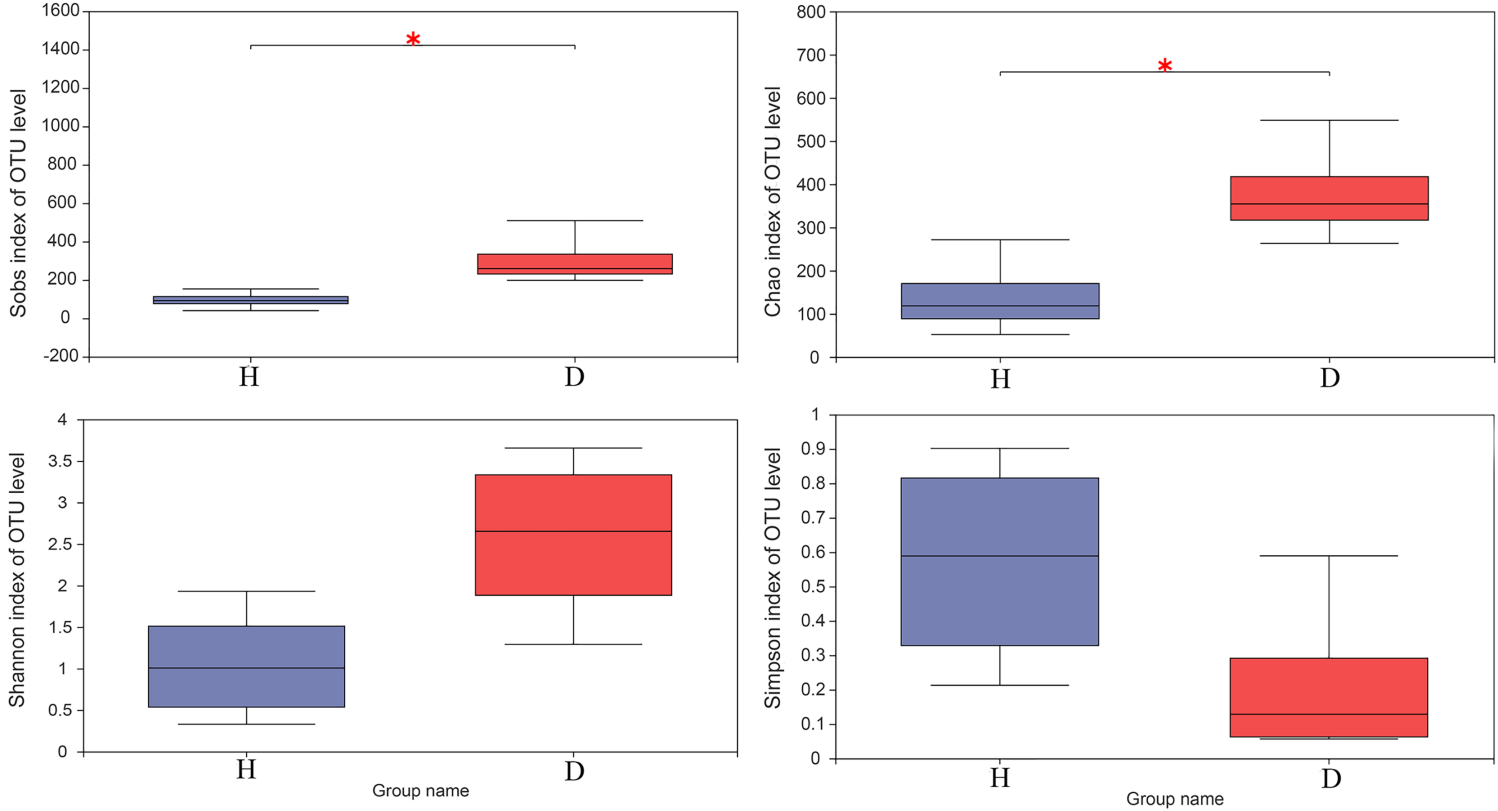
The Wilcoxon rank-sum test for alpha diversity indices of common kestrel samples, including Sobs, Chao, Shannon and Simpson indices. This graph displays the significant differences between the two selected groups of samples. The horizontal axis represents the group names, while the vertical axis represents the range of indices for each group. H means healthy group while D means disease group. An asterisk (*) indicates a *p*-value less than 0.05, while two asterisks (**) indicate a *p*-value less than 0.01.

The results of the Wilcoxon rank-sum test of alpha diversity (Sobs, Chao1, Shannon and Simpson) between the healthy and injured groups are presented in Fig. 3. The results based on the Sobs and Chao1 showed that the healthy and the injured samples have a significant difference in microbial diversity (*p* < 0.05).

**Figure 4:**
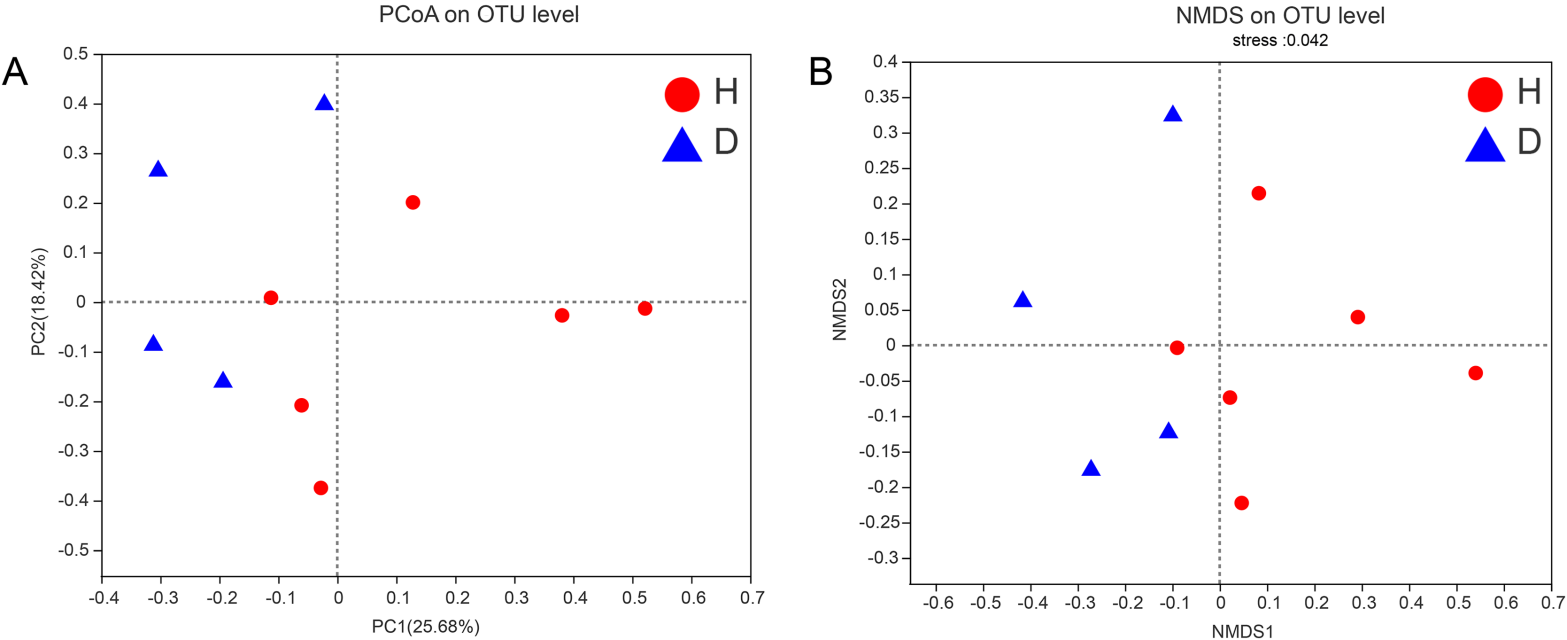
PCoA (A) and NMDS (B) of intestianal bacterial community structures of the common kestrels. Different colors or shapes of points represent samples from different groups. The closer the points of two samples are, the more similar their species composition is. PCoA and NMDS were generated with unweighted UniFrac distance. H means healthy group while D means disease group.

To assess the similarities or differences in the community composition between different groups, PCoA (unweighted UniFrac distance) (Fig. 4A) and NMDS (Fig. 4B) diagrams were generated. Similar to the results of the PERMANOVA (*p* < 0.05) described above, there was no clustering observed among the groups.

**Figure 5:**
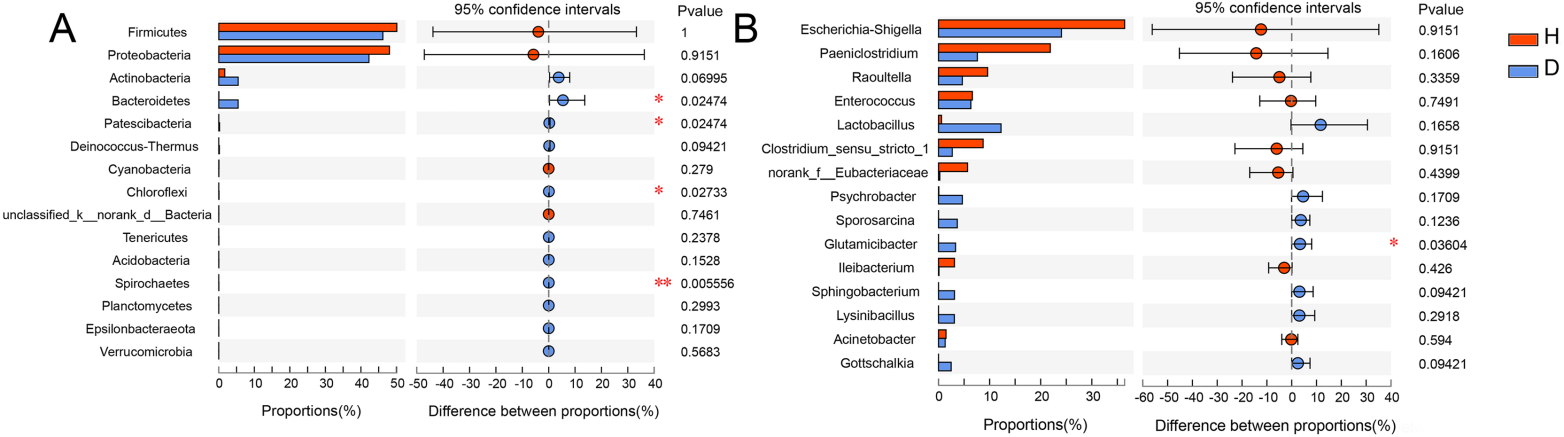
Wilcoxon rank-sum test bar plot at phylum (A) and genus (B) level. The X-axis represents different groups, with boxes of different colors representing different groups. The Y-axis represents the average relative abundance of a certain species in different groups. H means healthy group while D means disease group. An asterisk (*) indicates a *p*-value less than 0.05, while two asterisks (**) indicate a *p*-value less than 0.01.

To analyze the specific species, Wilcoxon rank-sum test bar plots at both the phylum (A) and genus (B) levels are shown in Fig. 5. The results indicated that the abundance of *Bacteroidetes* (*p* = 0.025), *Patescibacter* (*p* = 0.025), *Chloroflexi* (*p* = 0.027) and *Spirochaetes* (*p* = 0.006) were significantly different between healthy and injured common kestrels at the phylum level. While at the genus level, only *Glutamicibacter* (*p* = 0.036) showed significant difference.

## Discussion

The gut microbiota plays kinds of crucial roles in both humans and animals. With the rapid development of sequencing technology, related researches have become more and more in-depth. At present, sequencing data on the gut microbiota of birds remains limited, especially for raptors. As an indispensable part of the food chain, raptors are regarded as an important measure of the quality of the ecological environment.

Although the basic structure and composition of the fecal microbiota of an injured common kestrel was reported in our previous study (Guan et al., 2020), due to the difficulties of samples collection, the relevant data of healthy wild common kestrels has remained scarce.

In the present study, we characterized the composition and structure of the fecal microbiota for healthy and injured individuals and then made comparisons between the two groups.

Overall, the most predominant phyla of the fecal microbiota of the common kestrels in this study were *Firmicutes*, *Proteobacteria* and *Actinobacteria*, consistent with the previous findings on the fecal microbiota of injured wild common kestrel (Guan et al., 2020). At the phylum level, no significant differences in the relative abundances of the three phyla described above were observed between healthy and injured individuals. Previous results have shown that these three main phyla are dominant in most animal gut, and their actions may be related to the basic physiological activities of animals. In this study, there was no significant difference between the three phyla in the effects of injury and health on kestrel, because the effects of injury on individual intestinal microbes existed at a lower taxonomic unit level.

The relative abundance of *Bacteroidetes* in injured common kestrels (5.494%) was significantly higher (*p* = 0.025) than that observed in healthy birds (0.035%). Members of phylum *Bacteroidetes* have been found in many different habitats, where they exhibit a variety of biological functions, such as fermentation, absorption of nutrients (Thomas et al., 2011; Yang et al., 2021). *Bacteroidetes*, one of the most major phyla in the gut microbiota of common mammals and wild birds (Guan et al., 2016; Han et al., 2019; Wu et al., 2016, 2017; Zhao et al., 2017), plays crucial roles in degrading high molecular weight substance and carbohydrates secreted by the gut (Salyers et al., 1977; Thoetkiattikul et al., 2013).

Studies have found that genetically obese mice, even after being fed a low-fat, polysaccharide-rich diet, have higher *Firmicutes* and lower *Bacteroidetes* levels in their gut microbiota than lean mice fed the same diet (Ley et al., 2005). *Bacteroidetes* regardless of whether the diet was fat-limiting or carbohydrate-limiting, suggesting that changes in *Bacteroidetes* may be related to caloric intake (Ley et al., 2006). The same results were found in human fecal microorganisms.

In our study, the injured common kestrels were observed to generally eat less and be less active than the healthy due to poor physical conditions. Similarly, the abundance of *Bacteroidetes* increased when the obese mice lose their weight (Turnbaugh et al., 2006). As very diverse bacterial phyla, the proportion of *Bacteroidetes* and *Firmicutes* even exceeds 98% in the gut microbiota of mammals (Ley et al., 2006). A number of researches have reported that obese individuals have lower ratios of *Bacteroidetes* to *Firmicutes* (B/F) (Turnbaugh et al., 2009, 2006). Coincidentally, in our result, the healthy group showed the similar lower B/F value than the injured group. Hence, the higher relative abundance of *Bacteroidetes* may indicate the weight of the injured group should be lower than the healthy group. However, the ratios of Bacteroidetes to Firmicutes are also reported to be influenced by age factors. For example, a study on the intestinal microbial community of adults of different ages showed that older adults (0.6) have a lower ratio of Firmicutes to Bacteroidetes than young adults (10.9) (Mariat et al., 2009). Due to differences in age and gender, the weight range of the two groups of kestrels in our current study is relatively large. with the increasing number of rescued individuals, the composition of fecal microbiota in wild common kestrel would be more accurate. Besides, the weight changes between injured red kestrels and healthy red kestrels may be associated with medication treatment. During the rescue and treatment process, injured red kestrels often require medication to facilitate recovery and regain their health. These medications may include antibiotics, anti-inflammatory drugs, painkillers, among others. The use of these medications may have an impact on the weight of red kestrels. Certain medications may cause a decrease in appetite, weakened digestive function, or other metabolic changes in red kestrels, leading to weight fluctuations. Some medications may increase the appetite of red kestrels, resulting in increased food intake and weight gain. Additionally, certain medications may affect the metabolic rate of red kestrels, influencing energy expenditure and storage, thus impacting weight. Therefore, when comparing weight changes between injured red kestrels and healthy red kestrels, we should consider the potential impact of medication treatment. By analyzing the relationship between medication treatment and weight changes, we can gain a better understanding of the influence of medication on red kestrel weight and further optimize rescue and treatment strategies to facilitate their recovery and well-being. However, it is important to note that this hypothesis is based on speculative possibilities and requires further research to validate and explore the exact relationship between weight changes in injured red kestrels and medication treatment.

Although some members that belong to *Bacteroidetes* playing beneficial role in many respects for host, other genera, such as *Riemerella* and *Ornithobacterium*, would even cause septicemia and respiratory tract infections in birds (Segers et al., 1993; Vandamme et al., 1994). Furthermore, a large number of *Bacteroidetes* strains have been isolated from blood, urine, infected wounds and feces (Hugo et al., 1999). Therefore, we hypothesized that another reason for the significant difference in the relative abundance of *Bacteroidetes*, despite the strict requirements of BRRC for aseptic treatment, was the infected wounds of the injured common kestrels.

*Patescibacteria* was also another phylum that showed a significant difference (*p* = 0.025) between healthy (0.001%) and injured (0.226%) common kestrels. As a superphylum, *Patescibacteria* is regarded as an opportunistic or saprophytic colonizer that could reduce metabolic capabilities and participate in the hydrogen production, sulfur cycling, anaerobic methane oxidation (Peura et al., 2012; Wrighton et al., 2012; Zhang et al., 2020). The high abundance of *Patescibacteria* was reported to be relevant with high arsenic and metal content (Reis et al., 2016). For birds, *Patescibacteria* were also having detected in the gut microbiota of chicken (van Kuijk et al., 2020) and turkeys (Kursa et al., 2020). However, the injured and healthy individuals in our study were raised in the exactly same living environment. Consequently, the high metal content was not the cause of the significant differences in this phylum of the common kestrels we rescued in BRRC. Meanwhile, *Patescibacteria* was also closely correlated with the issues decay with the specific anoxic conditions. Hence, similar to the possible cause of the high relative abundance of *Bacteroidetes* in the injured group, we inferred that that the higher abundance of *Patescibacteria* in the injured group was caused by the wounds of common kestrels. The high relative abundance of *Patescibacteria* should be paid enough attention which may be the characterization of potential pathogens for birds, especially raptors.

The third phylum exhibiting a significant difference (*p* = 0.027) in healthy (0.004%) and injured (0.067%) common kestrels was *Chloroflexi*. *Chloroflexi* is a common dominant phylum in kinds of birds, such as Jankowski’s Bunting (*Emberiza jankowskii*) (Shang et al., 2020), Hooded Crane (*Grus monacha*) (Fu et al., 2020) and house sparrows (*Passer domesticus*) (Gadau et al., 2019). On the one hand, some researches indicated that *Chloroflexi* plays important role in the process of carbohydrate degradation (Ariesyady, Ito & Okabe, 2007; Sekiguchi et al., 2001). On the other hand, the study of mockingbirds showed that the bacteria from the phylum *Chloroflexi* may have induced an antibody-mediated immune response against infection and antibody levels were positively correlated with the relative abundance of this phylum (Knutie, 2018). Although the specific metabolic pathways of the members that belong to *Chloroflexi* were not clear completely until now, the results of our study indicated that the infection should be the most crucial reason for the significant higher abundance of *Chloroflexi* in the injured common kestrels. Additionally, the relative abundance of *Spirochaetes* was higher in injured common kestrels than in healthy group (*p* = 0.006). Studies have also shown that *Spirochaetes* cause dementia and are linked to the pathogenesis of Alzheimer’s disease (Miklossy, 2011). Most bacteria in *Spirochaetes* can live independently and are usually anaerobic. It is highly diverse in its pathogenicity and on the microecological scale of its existence, as well as in its molecular biological characteristics, including guanine-cytosine content and its genome size. In this phylum, *Leptospira* can cause leptospirosis, while *Borrelia burgdorferi*, *B. garinii* and *B. afzelii* can cause Lyme disease (Anderson et al., 1986; Anderson & Magnarelli, 1984). Moreover, it is worth noting that the songbirds who has a special ability to act as a host for transmission, plays important roles in the epidemiology of Lyme disease (Scott et al., 2010). The remarkable difference of relative abundance in *Spirochaetes* was observed between injured and healthy common kestrels, which suggested that for rescuing rare wild birds, more attention should be paid for their real physical condition, such as the test for Lyme disease and other infectious diseases, as well as the conventional trauma. Therefore, the result of our present study indicated that the occurrence and the relative abundance of *Spirochaetes* are of great significance for monitoring epidemiology of birds.

However, at the genus level, the *Glutamicibacter* was the only genus which significantly higher (*p* = 0.036) in injured common kestrel than in healthy group. The genus *Glutamicibacter* belongs to the family *Micrococcaceae* and are believed to play an important role in many ecosystems and affect human well-being (Yamamoto et al., 2016). The representatives of genus *Glutamicibacter* were found to be the basic composition of gut microbiota in many wild animal, such as crocodile lizard (Tang et al., 2020), grass carp (Huang et al., 2018) and grasshopper (Wang et al., 2020). Interestingly, although there are few clinical cases, the species G. *creatinolyticus* has been reported to be associated with infectious processes, such as bacteremia and urinary tract infection (Hou et al., 1998; Yamamoto et al., 2016). These reports are consistent with the above results and hypotheses based on significant differences in relative abundance of *Bacteroidetes*. In other words, the significant higher relative abundance of genus *Glutamicibacter* indicated that the injured common kestrels may suffer certain infections and inflammations during the process of rescue by BRRC. Although the injured common kestrels in the BRRC did not have other infections or severe inflammations, wildlife disease surveillance capabilities of organizations still need to be developed and explored, which puts more stringent requirements on wildlife rescue practitioners. Therefore, one of the next steps is to improve the efficiency of disease surveillance in wild birds, as well as their diagnostic capabilities.

Meanwhile, it should be noted that there are still limitations in our present study. First, although the number of common kestrels rescued by BRRC are limited, more fecal samples and gastrointestinal contents, if possible, should be collected from more different common kestrel individuals. Moreover, age and sex also should be considered as important influence factor that would affects the gut microbiota of host. Second, metagenomic sequencing should be added into the next study to explore the mechanism and function of gut microbiota in common kestrel more deeply.

There is a close relationship between the rescue of wild rare bird species and gut microbiota diversity, which is a highly focused research field. The gut microbiota plays a crucial role in the health and survival of the host. The rescue of wild bird species involves the rescue, treatment, and rehabilitation of injured, sick, or displaced birds, which may have an impact on their gut microbiota community. Environmental factors, dietary habits, and host health status can all have an impact on the composition and functionality of the gut microbiota. In the rescue of wild birds, factors such as dietary changes, medication treatments, and human contact can potentially affect the diversity of the avian gut microbiota. By studying the gut microbial communities of wild birds, we can gain a better understanding of their health status, nutrient intake, and adaptability to environmental changes. In addition, studies on gut microbial diversity can provide valuable information for wildlife bird rescue programs. By comparing the gut microbial communities of rescued birds with those of wild populations, we can assess the impact of the rescue process on avian gut health and propose improvement measures. This helps optimize rescue and rehabilitation strategies, enhancing the survival and adaptability of wild birds.

## Supplemental Information

10.7717/peerj.15789/supp-1 Supplemental Information 1 The information of common kestrels.

10.7717/peerj.15789/supp-2 Supplemental Information 2 Rarefaction curves. The curves reflect the rationality of sequencing data size and abundance of species in samples.

## Additional Information and Declarations

### Data Availability

The following information was supplied regarding data availability: The raw data are available at the NCBI Sequence Read Archive (SRA): SRP293621.
