# Supplementary material for: Comparative analysis of the fecal microbiota of healthy and injured common kestrel (Falco tinnunculus) from the Beijing Raptor Rescue Center
Source: PeerJ. 2023 Aug 22;11:e15789. doi: 10.7717/peerj.15789 (PMC10452619; doi:10.7717/peerj.15789)
Supplement: Supplemental Information 1 [file peerj-11-15789-s001.docx]

**Table S1. The information of common kestrel**

| **Group** | **Sample** | **physical condition** | Age | **Weight** | **Sex** |
| --- | --- | --- | --- | --- | --- |
| H | H1 | Healthy | 8-12 | 180-300 | Male |
|  | H2 | Healthy | 8-12 | 180-300 | Female |
|  | H3 | Healthy | 8-12 | 180-300 | Male |
|  | H4 | Healthy | 8-12 | 180-300 | Male |
|  | H5 | Healthy | 8-12 | 180-300 | Male |
|  | H6 | Healthy | 8-12 | 180-300 | Female |
| D | D1 | Injured | 8-12 | 180-300 | Male |
|  | D2 | Injured | 8-12 | 180-300 | Male |
|  | D3 | Injured | 8-12 | 180-300 | Male |
|  | D4 | injured | 8-12 | 180-300 | Female |
