# Supplementary figures and images for: Comparative analysis of the fecal microbiota of healthy and injured common kestrel (Falco tinnunculus) from the Beijing Raptor Rescue Center
Source: PeerJ. 2023 Aug 22;11:e15789. doi: 10.7717/peerj.15789 (PMC10452619; doi:10.7717/peerj.15789)

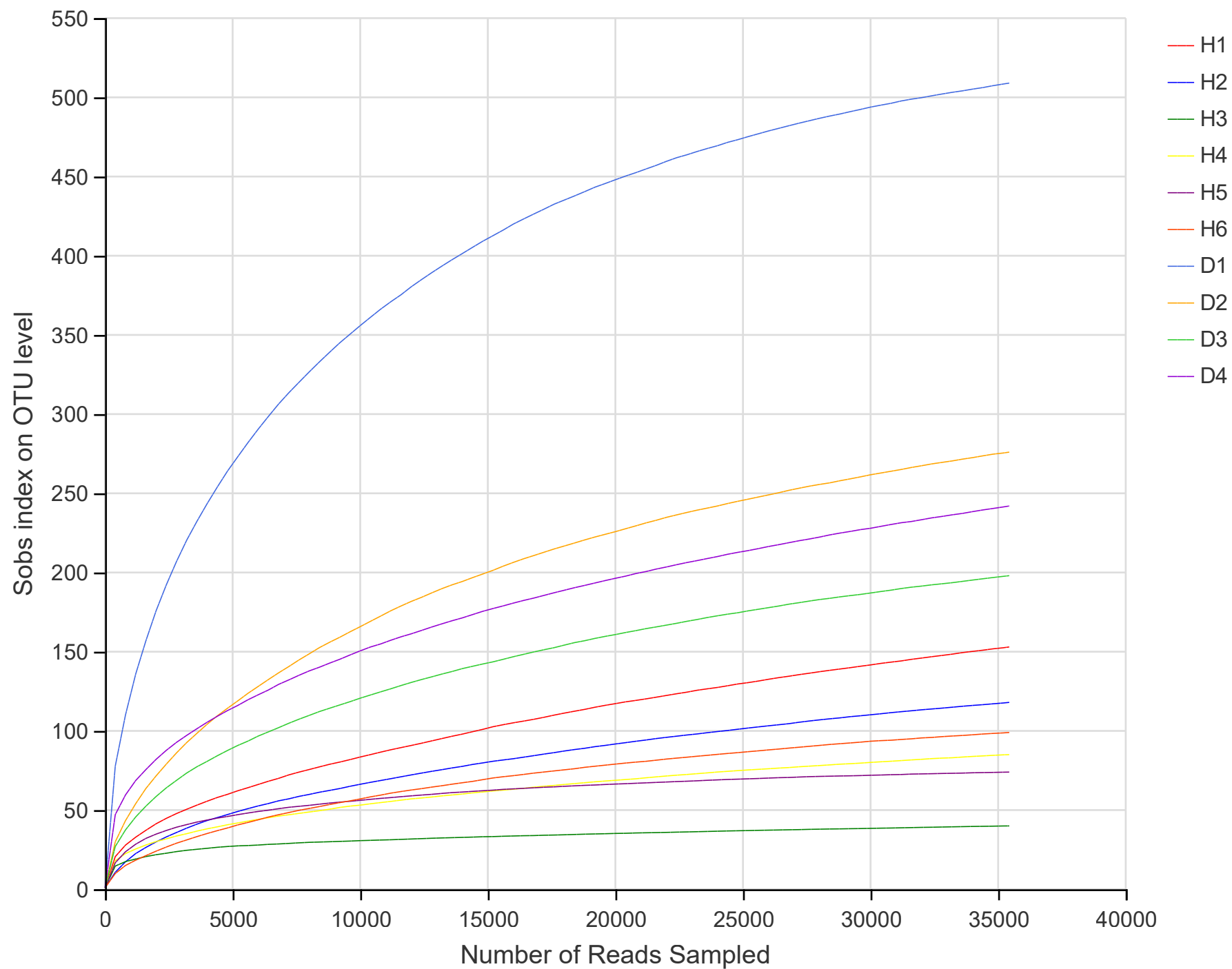

Supplement: Supplemental Information 2 — The curves reflect the rationality of sequencing data size and abundance of species in samples. [file peerj-11-15789-s002.pdf]
